# Thermoregulatory role of ghrelin in the induction of torpor under a restricted feeding condition

**DOI:** 10.1038/s41598-021-97440-y

**Published:** 2021-09-13

**Authors:** Takahiro Sato, Kanae Oishi, Daisuke Koga, Takanori Ida, Yusuke Sakai, Kenji Kangawa, Masayasu Kojima

**Affiliations:** 1 Division of Molecular Genetics, Institute of Life Science, Kurume University, 67 Asahi-machi, Kurume, Fukuoka 830-0011 Japan; 2 Department of Microscopic Anatomy and Cell Biology, Asahikawa Medical University, Asahikawa, Hokkaido 078-8510 Japan; 3 Division for Searching and Identification of Bioactive Peptides, Department of Bioactive Peptides, Frontier Science Research Center, University of Miyazaki, Miyazaki, 889-1692 Japan; 4 Institute of Animal Experimentation, Kurume University School of Medicine, 67 Asahi-machi, Kurume, Fukuoka 830-0011 Japan; 5 Department of Biochemistry, National Cerebral and Cardiovascular Center Research Institute, Suita, 565-8565 Japan

**Keywords:** Metabolism, Physiology, Endocrinology

## Abstract

Ghrelin, a circulating orexigenic hormone secreted from the stomach, stimulates appetite and food intake by activating the hypothalamic arcuate nucleus. Administration of exogenous ghrelin exerts anabolic effects, causing weight gain, increased adiposity, and decreased metabolism. Body temperature (BT), which is determined by the balance of heat production and heat loss, must be strictly regulated to maintain proper cellular function and metabolism. However, the role of ghrelin in thermoregulation remains unclear. In this study, we found that ghrelin was essential for decreasing BT when mice are placed under calorie restriction. Elevated ghrelin concentrations induced by fasting correlated with significant decreases in BT, a hibernation-like state called torpor. Ghrelin-deficient (*Ghrl*^*−/−*^) animals could not enter torpor. The BT of *Ghrl*^*−/−*^ mice also remained high under restricted feeding, but the animals gradually entered precipitous hypothermia, indicating thermoregulatory impairment. These effects of ghrelin on thermoregulation were the result of suppression of sympathetic nervous system activity input to brown adipose tissue; in the absence of ghrelin, it was not possible to suppress uncoupling protein 1 (*ucp1*) expression and decrease BT in low-energy states. Together, these findings demonstrate that ghrelin is an essential circulating hormone involved in lowering BT.

## Introduction

Total energy consumption is the sum of basal metabolism, behavioral activity, and diet-induced thermogenesis. Changes in these components lead to heat production or heat loss, reflected by alteration in body temperature (BT). Therefore, under low-energy states such as starvation, proper BT regulation is necessary to prevent the waste of energy. Biological factors related to this regulation can detect the energy state and modulate energy consumption accordingly. Among the hormones and neuropeptides that play integral roles in the regulation of energy metabolism, ghrelin is a candidate mediator of thermoregulation under low-energy states^1^. Fasting is associated with elevated ghrelin mRNA expression and high plasma ghrelin concentration^2–5^.

Previously, several studies have investigated whether ghrelin is involved in thermoregulation, including regulation of torpor. In small animals like mice, torpor, a hibernation-like state associated with a significant fall in BT and decreased metabolic rates, is induced by fasting. Leptin, an anorexigenic hormone produced by adipocytes, transmits a signal that promotes entry into torpor in mice^6,7^. Because ghrelin physiologically antagonizes leptin, this led us to hypothesize that ghrelin may be involved in the regulation of torpor.

In this study, we sought to clarify whether ghrelin is required for induction of torpor in severe low-energy states and to elucidate the thermoregulatory mechanism of ghrelin.

## Results

### Ghrelin-deficient mice do not enter torpor

**Figure 1:**
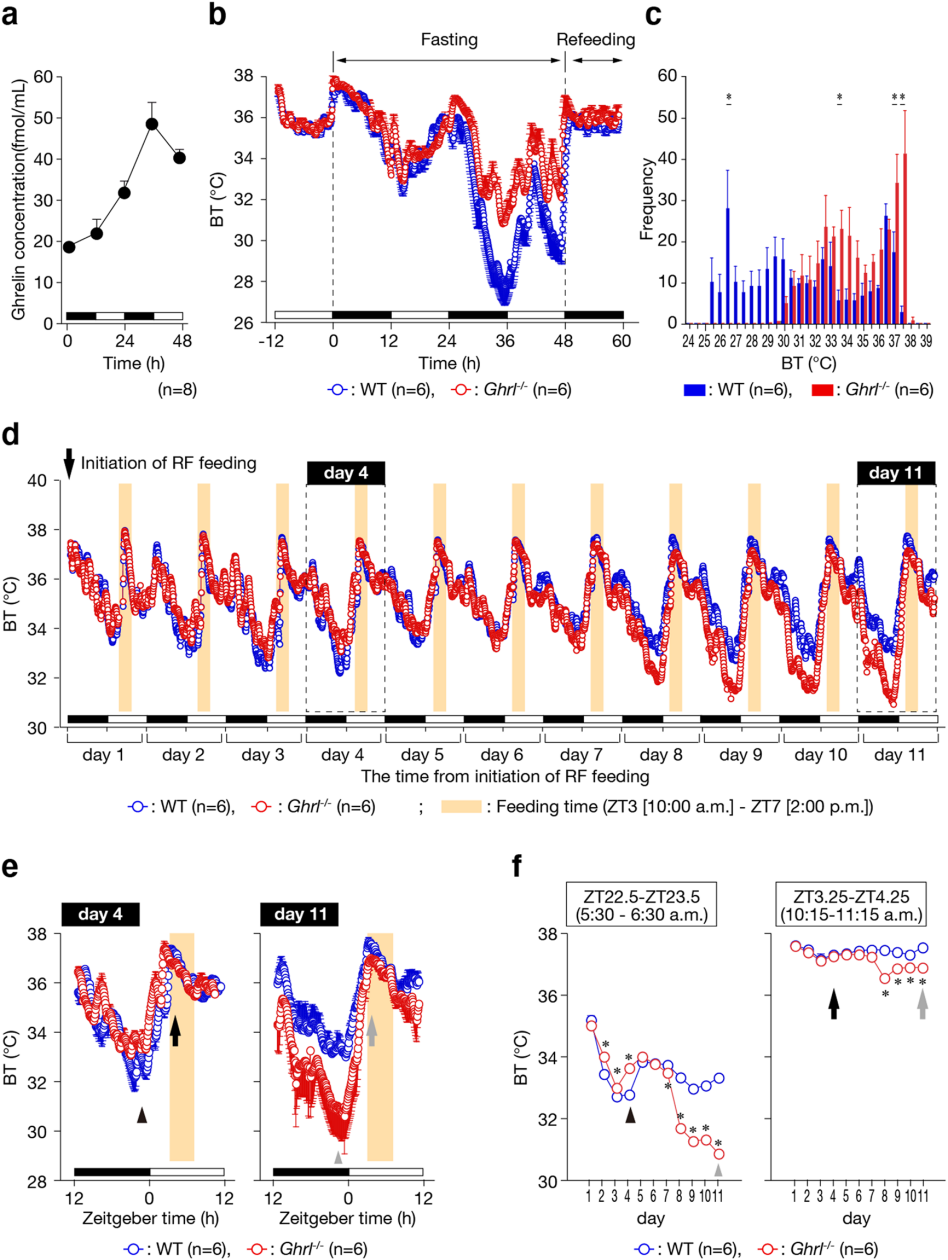
Lack of torpor induction in *Ghrl*^*−/−*^ mice. (**a**) Plasma acyl ghrelin concentrations in fasting mice. Plasma was collected every 12 h. *P < 0.05 (0 h [n = 8] vs. 12, 24, 36, and 48 h [n = 8 for each group]). (**b**) Relatively high BT was observed in *Ghrl*^*−/−*^ mice (n = 6) at times when torpor was induced in WT (n = 6). BT was measured every 5 min, and all data were averaged. (**c**) Frequency distribution of BT during the fast. Data were collected between 24 and 48 h after fasting initiation. *P < 0.05 (WT mice [n = 6] vs. *Ghrl*^*−/−*^ mice [n = 6]). Error bars in all figures indicate S.E. (**d**) Characteristic BT changes in *Ghrl*^*−/−*^ mice during restricted feeding. Mice were given food for 4 h from ZT3 (10:00 a.m.) to ZT7 (2:00 p.m.) per day. (**e**) Representative data during restricted feeding. Due to ghrelin deficiency, core BT was high during the fasting period on day 4, but low during the fasting period on day 11. (**f**) BT variations in the hunger and satiety periods. *Left panel* (a hunger period): ZT22.5 (5:30 a.m.)–ZT23.5 (6:30 a.m.); this hour represents the time that is the most hypothermic state. *Right panel* (a satiety period): ZT3.25 (10:15 a.m.)–ZT4.25 (11:15 a.m.); this hour is the time that reflects the BT of an early phase during the feeding period. *P < 0.05 (WT mice [n = 6] vs. *Ghrl*^*−/−*^ mice [n = 6]). Error bars in (**e**) and (**f**) indicate S.E.

We performed telemetric analysis of wild-type (WT) and ghrelin-deficient (*Ghrl*^*−/−*^; Supplymentary Fig. 1) mice to monitor BT during fasting, when ghrelin secretion is physiologically elevated. As previously reported, plasma ghrelin concentrations gradually increased, peaking 36 h after fasting initiation (Fig. 1a). During low-energy states such as fasting, small animals lower BT and metabolic rate to conserve energy. This state, called torpor, is a hibernationlike hypothermic condition that generally lasts several hours. In WT mice, deep torpor was induced within 24–48 h after the initiation of fasting, as signified by decreases in minimum core BT to 26.9 ± 0.8 °C (Fig. 1b, c). By contrast, *Ghrl*^*−/−*^ mice did not enter torpor even under the fasting condition, when high levels of plasma ghrelin would normally be observed. During this period, *Ghrl*^*−/−*^ mice had an average minimum core BT of 31.5 ± 0.3 °C (Fig. 1b, c). In addition, there were no differences in hormones and blood parameters that could alter ghrelin secretion between WT and *Ghrl*^*−/−*^ mice (Supplementary Fig. 2). These results indicate that ghrelin is essential for inducing torpor to decrease BT.

We next carried out a restricted feeding experiment to observe the adaptation of BT to the low-energy state. Under restricted feeding conditions, the mice were given food for only 4 h a day (ZT3–ZT7 [10:00 a.m.–2:00 p.m.]). In both WT and *Ghrl*^*−/−*^ mice, restricted feeding induced a hypothermic state with a minimal value of BT approximately 4 h before feeding time (Fig. 1d, e). However, in accordance with the results obtained under fasting conditions, the BT of *Ghrl*^*−/−*^ mice was higher in the hunger period of dark phase (ZT22.5–ZT23.5 [5:30 a.m.–6:30 a.m.]) from day 2 to day 4 (Fig. 1e, f). On the contrary, BT dramatically decreased in *Ghrl*^*−/−*^ mice from day 7 to day 11 (Fig. 1e, f). Furthermore, ghrelin deficiency also induced a decrease in BT from day 8 to day 11 during the satiety period (ZT3.25–ZT4.25 [10:15 a.m.–11:15 a.m.]) (Fig. 1e, f). These results suggest that thermoregulation fails in *Ghrl*^*−/−*^ mice in a low-energy state.

### Ghrelin deficiency fails to lower resting temperature

**Figure 2:**
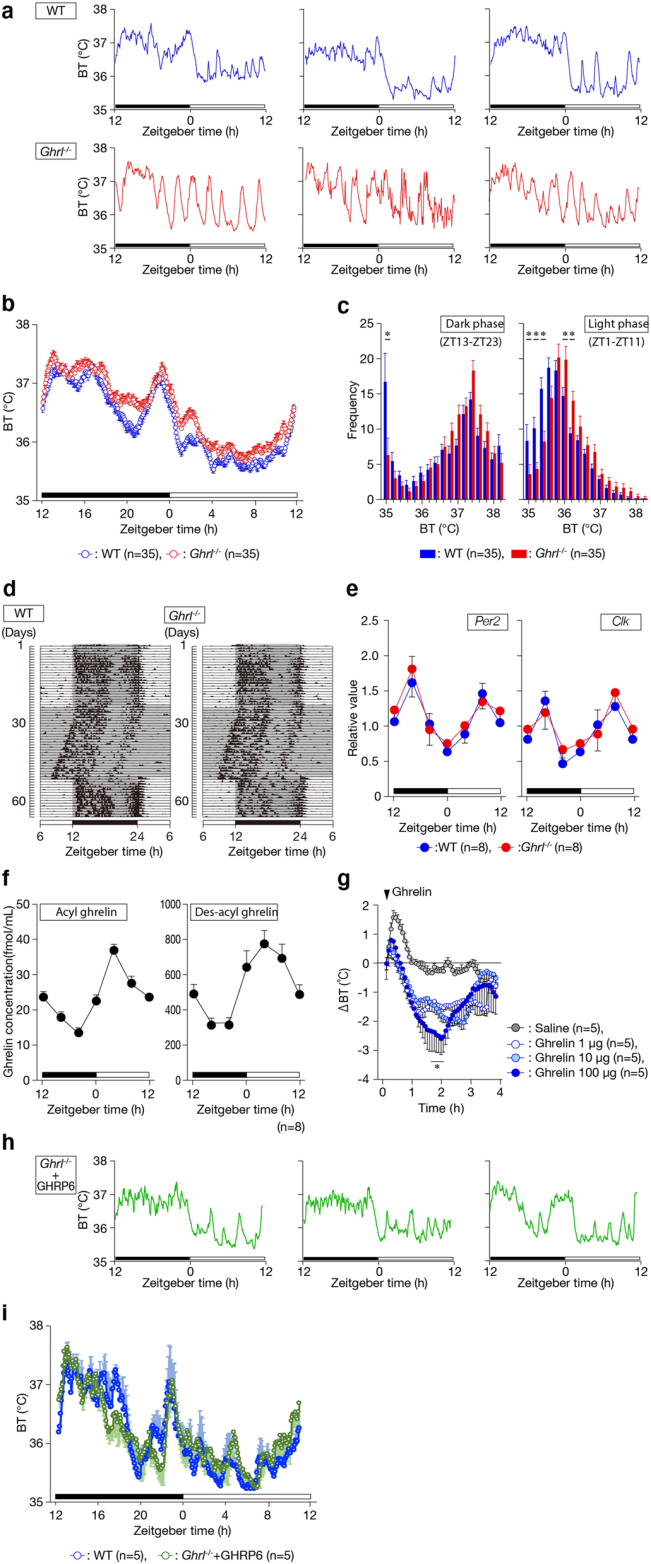
Ghrelin is necessary for lowering BT. (**a**) Telemetric analysis of core BT in individual WT and *Ghrl*^*−/−*^ mice. BT was measured every 5 min for 24 h, and data from all mice were averaged. (**b**) Diurnal rhythm of mean BT in *Ghrl*^*−/−*^ mice. WT mice [n = 35] vs. *Ghrl*^*−/−*^ mice [n = 35]. (**c**) Frequency distribution of BT during the light (8:00 a.m.–5:00 p.m.) and dark phases (8:00 p.m.–6:00 a.m.). (Because there were large BT variations around the periods between light and dark phases, these periods are excluded.) The bin width of the frequency distribution was 0.2 °C. A phase shift to high BT was observed. *P < 0.05 (WT mice [n = 35] vs. *Ghrl*^*−/−*^ mice [n = 35]). (**d**) Locomotor activity of WT and *Ghrl*^*−/−*^ mice. Periodograms analyzing locomotor activity reveal normal circadian rhythms in *Ghrl*^*−/−*^ mice under light–dark (LD) and dark–dark (DD) conditions. The actogram was created in Adobe Illustrator (ver. 25.0.1, https://www.adobe.com/jp/products/illustrator.html). (**e**) Circadian clock genes of *Ghrl*^*−/−*^ mice. Expression levels of *Per2* and *Clk* did not differ between WT and *Ghrl*^*−/−*^ mice throughout the day. (WT [n = 8] vs. *Ghrl*^*−/−*^ [n = 8] mice). (**f**) Both plasma acyl and des-acyl ghrelin concentrations exhibited circadian rhythms. Plasma was collected every 4 h. (**g**) BT decreased following intraperitoneal injection of rat ghrelin. Injection was carried out on ZT3 using three ghrelin concentrations. Two hours later, BT reached a nadir. *P < 0.05 (Saline [n = 6] vs. 1, 10, and 100 µg ghrelin [n = 6 for each group]). Error bars in all figures indicate S.E. (**h**) Telemetric analysis of core BT in three individual *Ghrl*^*−/−*^ mice equipped with an osmotic minipump filled with GHRP-6. (**i**) Diurnal rhythm of mean BT in *Ghrl*^*−/−*^ mice treated with GHRP-6. WT mice [n = 6] vs. *Ghrl*^*−/−*^ mice [n = 6].

In nocturnal animals such as mice, BT exhibits a clear circadian rhythm, peaking in the dark phase and reaching a nadir in the light phase. Hence, we examined circadian BT in WT and *Ghrl*^*−/−*^ mice using telemetry. Although both WT and *Ghrl*^*−/−*^ mice had high BT in the dark phase and low BT in the light phase (Fig. 2a, b), individual *Ghrl*^*−/−*^ mice exhibited arrhythmic peaks primarily in the light phase. However, there was considerable individual variability (Fig. 2a). In addition, because BT was slightly higher in *Ghrl*^*−/−*^ mice than in WT mice (Fig. 2a,b), we carried out a frequency analysis at intervals of 0.2 °C. The results clearly showed that BT in *Ghrl*^*−/−*^ mice was high in the light phase, but not in the dark phase (Fig. 2c), in comparison with WT mice. These results show that ghrelin-deficient mice are unable to decrease their resting BT.

### Ghrelin-deficient mice cannot maintain diurnal rhythm of body temperature

Next, to determine whether the diurnal rhythm is maintained in *Ghrl*^*−/−*^ mice, we analyzed the BT rhythm for 10 days (Table 1). Because BT is controlled by behavioral thermoregulation and autonomic thermoregulation, we first examined the circadian behavior of *Ghrl*^*−/−*^ mice. The rhythmicity of circadian activity in *Ghrl*^*−/−*^ mice was normal under both a 12-h light / 12-h dark (LD) cycle and a constant dark (DD) condition (Fig. 2d). In fact, the same free-running period was observed under DD conditions (WT: 23.8 ± 0.1, GKO: 23.8 ± 0.2; n = 6 in each group). Consistent with this observation, the circadian expression of *Period2 (Per2)* and *Clock (Clk)* in the suprachiasmatic nucleus (SCN) in *Ghrl*^*−/−*^ mice was also normal (Fig. 2e). Thus, in *Ghrl*^*−/−*^ mice, the biological clock in the SCN was normal.

**Table 1:** The analysis of BT for 10 days in *ghrl*^*−/−*^ mice.

	WT	*Ghrl*^*−/−*^	*p* value	Significance
**Mean (°C)**				
Light	35.8 ± 0.04	36.0 ± 0.06	0.0008	***
Dark	37.2 ± 0.06	37.1 ± 0.05	0.1789	
Light mean—Dark mean	1.40 ± 0.06	1.08 ± 0.06	0.0031	**
**AUC (a.u.)**				
Light	430.5 ± 0.76	433.4 ± 0.55	0.0086	**
Dark	446.5 ± 1.18	444.5 ± 0.95	0.2084	
**Highest reading (°C)**				
Light	38.4 ± 0.06	38.4 ± 0.06	0.8397	
Dark	38.4 ± 0.05	38.4 ± 0.06	0.9287	
**Lowest reading (°C)**				
Light	34.5 ± 0.07	34.8 ± 0.09	0.0209	*
Dark	35.4 ± 0.11	35.5 ± 0.10	0.3498	
%rhythm (%)	72.6 ± 0.98	63.9 ± 3.10	0.0411	*

Light (Light phase), 7:00–19:00; Dark (Dark phase), 19:00–7:00. AUC, area under curve. The asterisks of significance column means.

*p < 0.05; **p < 0.01, and ***p < 0.001.

However, the rhythmicity of BT in *Ghrl*^*−/−*^ mice was diminished; the difference in diurnal range of BT (dark phase mean − light phase mean) was significantly smaller in *Ghrl*^*−/−*^ mice (Table 1). Accordingly, the area under the curve (AUC) of the light phase was also larger in *Ghrl*^*−/−*^ mice (Table 1). The lowest reading (i.e., minimum BT) in the light phase was higher in *Ghrl*^*−/−*^ mice than in WT mice, although no differences were observed in the dark phase (Table 1). On the other hand, we observed no difference between WT mice and *Ghrl*^*−/−*^ mice in the highest reading (i.e., maximum BT) in either the light phase or the dark phase (Table 1). Moreover, 10-day analyses clearly revealed the abnormal BT rhythm in *Ghrl*^*−/−*^ mice (WT mice: 72.6 ± 0.98%, *Ghrl*^*−/−*^ mice: 63.9 ± 3.10%) (Table 1). Thus, deficiency of *Ghrl* caused an increase in BT in the light phase, as well as an abnormality in BT rhythm. Based on these observations, we concluded that ghrelin exerts thermoregulatory effects by lowering BT and maintaining daily BT patterns.

### Ghrelin induces hypothermia

Because BT was higher in *Ghrl*^*−/−*^ mice than in WT mice, we investigated the correlation between BT and ghrelin secretion. Both acyl and des-acyl ghrelin levels in WT mice were lower in the dark and higher in the light (Fig. 2f). Thus, plasma ghrelin concentrations were inversely correlated with BT. In fact, intraperitoneal administration of ghrelin to WT mice induced a hypothermic state, with BT reaching a nadir about 2 h later; this pattern held for all doses tested (Fig. 2g). Furthermore, *Ghrl*^*−/−*^ mice treated for 2 weeks with GHRP-6, a ghrelin receptor agonist, reduced BT rhythm abnormalities and restored diurnal rhythms similar to those of the WT mice shown in Fig. 2a (Fig. 2h). Moreover, GHRP-6 treatment of *ghrl*^*−/−*^ mice caused diurnal changes in BT to be similar to those of WT mice (Fig. 2i). Thus, the diurnal rhythm of BT was inversely correlated with plasma ghrelin concentration, and ghrelin had a hypothermic effect.

### The effect of ghrelin thermoregulation is mediated by the arcuate nucleus and vagus nerve

The hypothalamic arcuate nucleus is one of the target tissues of ghrelin involved in stimulation of appetite and feeding behavior. In addition, the vagus nerve transmits a neural ghrelin signal from peripheral organs to the central nervous system. Hence, to examine the effects of these organ tissues on BT regulation, we destroyed the arcuate nucleus and the vagus nerve.

**Figure 3:**
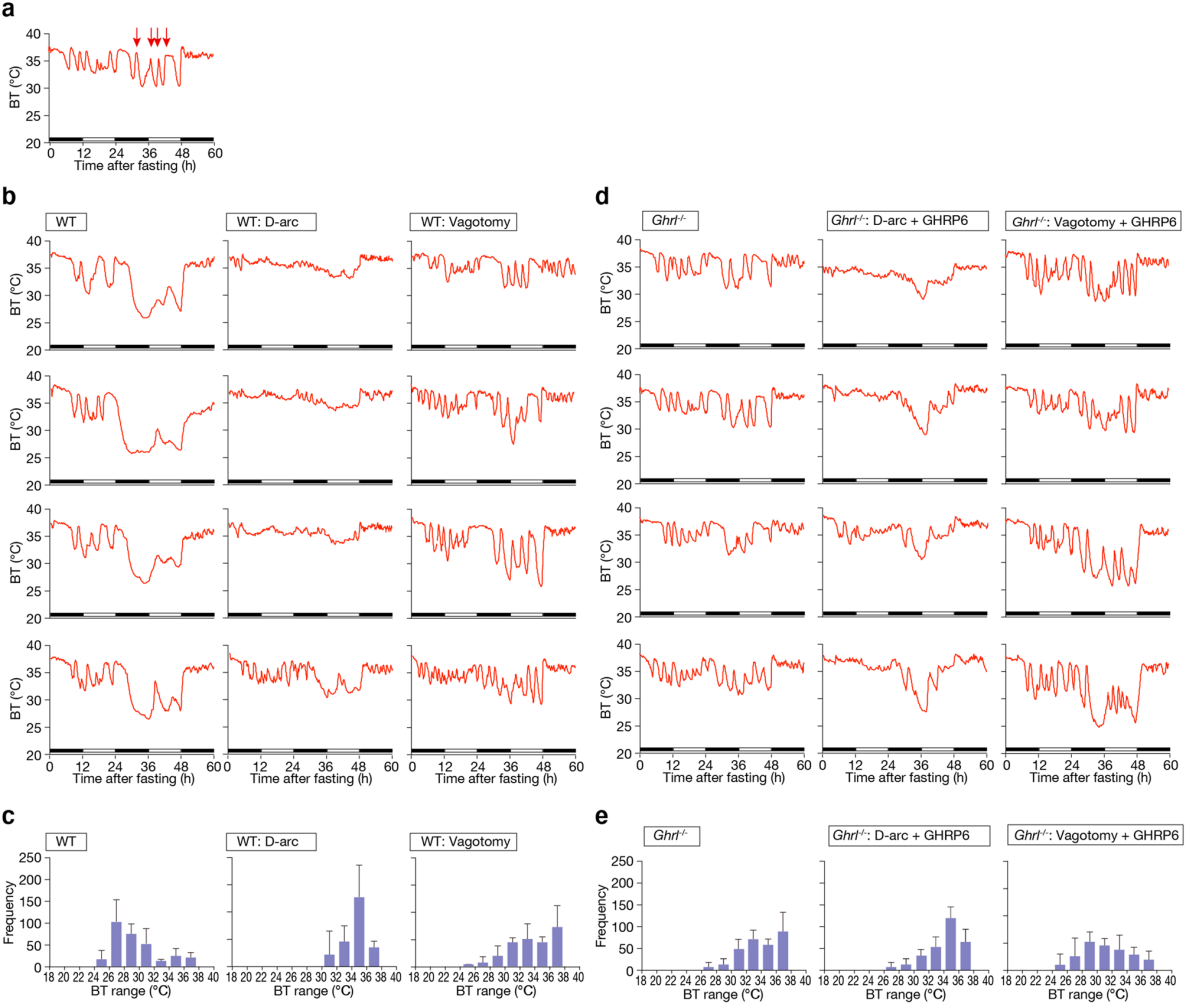
Ghrelin pathway and function. (**a**) Fluctuation of BT is indicated by the red arrows. As shown in (**b**,**d**), the fluctuation of BT is slow, cycling with a periodicity of 2–3 h. (**b**) Role of the arcuate nucleus and vagus nerve in thermoregulation during fasting in C57BL/6 J mice. Each graph shows the results for each individual; mice were re-fed 48 h after the start of fasting. Destruction of the arcuate nucleus suppressed the decrease in BT during fasting, and vagotomy destabilized the BT rhythm. D-arc: arcuate nucleus destruction. (**c**) Data from (**b**), shown as a histogram from 24 to 48 h after the start of fasting, during which torpor occurs. (**d**) Role of the arcuate nucleus and vagus nerve in thermoregulation during fasting in *Ghrl*^*−/−*^ mice treated with an osmotic minipump filled with GHRP-6. Each graph shows the results for one individual. Mice were re-fed 48 h after the start of fasting. Destruction of the arcuate nucleus in *Ghrl*^*−/−*^ mice treated with an osmotic minipump filled with GHRP-6 resulted in stable BT and minimal hypothermia. Vagotomy in *Ghrl*^*−/−*^ mice treated with an osmotic minipump filled with GHRP-6 caused a large decrease in BT with occasional peaks of high BT. D-arc: arcuate nucleus destruction. (**e**) Data from Fig. 3d are shown as a histogram from 24 to 48 h after the start of fasting.

As described above, ablation of ghrelin results in loss of torpor induction and fluctuation of BT. The latter is shown by the red arrows in Fig. 3a. The fluctuation of BT was slow, cycling with a period of 2–3 h. The arcuate nucleus was necessary for torpor induction, whereas the vagus nerve was necessary for the stability of BT.

The reduction of BT during fasting, i.e., the induction of torpor, was not observed in mice after destruction of the arcuate nucleus (D-arc) (Fig. 3b,c). On the other hand, mice subjected to vagotomy retained torpor induction, although they exhibited severe fluctuation in BT (Fig. 3d,e). In the vagotomized mice, the cycles of BT fluctuation had periods of 24–48 h, almost the same as in *Ghrl*^*−/−*^ mice.

We next investigated whether loss of torpor induction and fluctuation of BT would be rescued by administration of ghrelin to the arcuate nucleus or the vagus nerve. To this end, we destroyed the arcuate nucleus or performed vagotomy in *Ghrl*^*−/−*^ mice and administered ghrelin. Administration of ghrelin to D-arc *Ghrl*^*−/−*^ mice stimulated the vagus nerve, whereas administration of ghrelin to vagotomized *Ghrl*^*−/−*^ mice stimulated the arcuate nucleus. We used GHRP-6, a ghrelin receptor agonist, as a ghrelin mimetic, because in this experiment we needed continuous administration of ghrelin for more than 48 h, and GHRP-6 is more stable than ghrelin.

With continuous administration of GHRP-6, the *Ghrl*^*−/−*^ mice in which the arcuate nucleus had been destroyed (the D-arc *Ghrl*^*−/−*^ mice) exhibited a mild decrease in BT during fasting. Because complete ablation of the arcuate nucleus is technically difficult, this result implies that the remnant of the arcuate nucleus was stimulated by exogenous GHRP-6, resulting in a slight decrease in BT (Fig. 3d,e). However, we observed no abnormality in the fluctuation of BT, as seen in *Ghrl*^*−/−*^ mice. Thus, it seems that exogenous GHRP-6 stimulated the intact vagus nerve to stabilize the fluctuation of BT. Together, these results indicate that ghrelin stabilizes the fluctuation of BT through activation of the vagus nerve.

By contrast, with continuous administration of GHRP-6, the vagotomized *Ghrl*^*−/−*^ mice exhibited a marked decrease in BT during fasting, although the fluctuation in BT was still observed (Fig. 3d,e). Because the arcuate nucleus of the vagotomized *Ghrl*^*−/−*^ mice was intact, this result indicates that the vagus nerve is necessary for maintaining the stability of BT.

Taken together, our findings demonstrate that during fasting, ghrelin lowers BT mainly via the arcuate nucleus and stabilizes BT via the vagus nerve.

### Ghrelin suppresses expression of Ucp1

**Figure 4:**
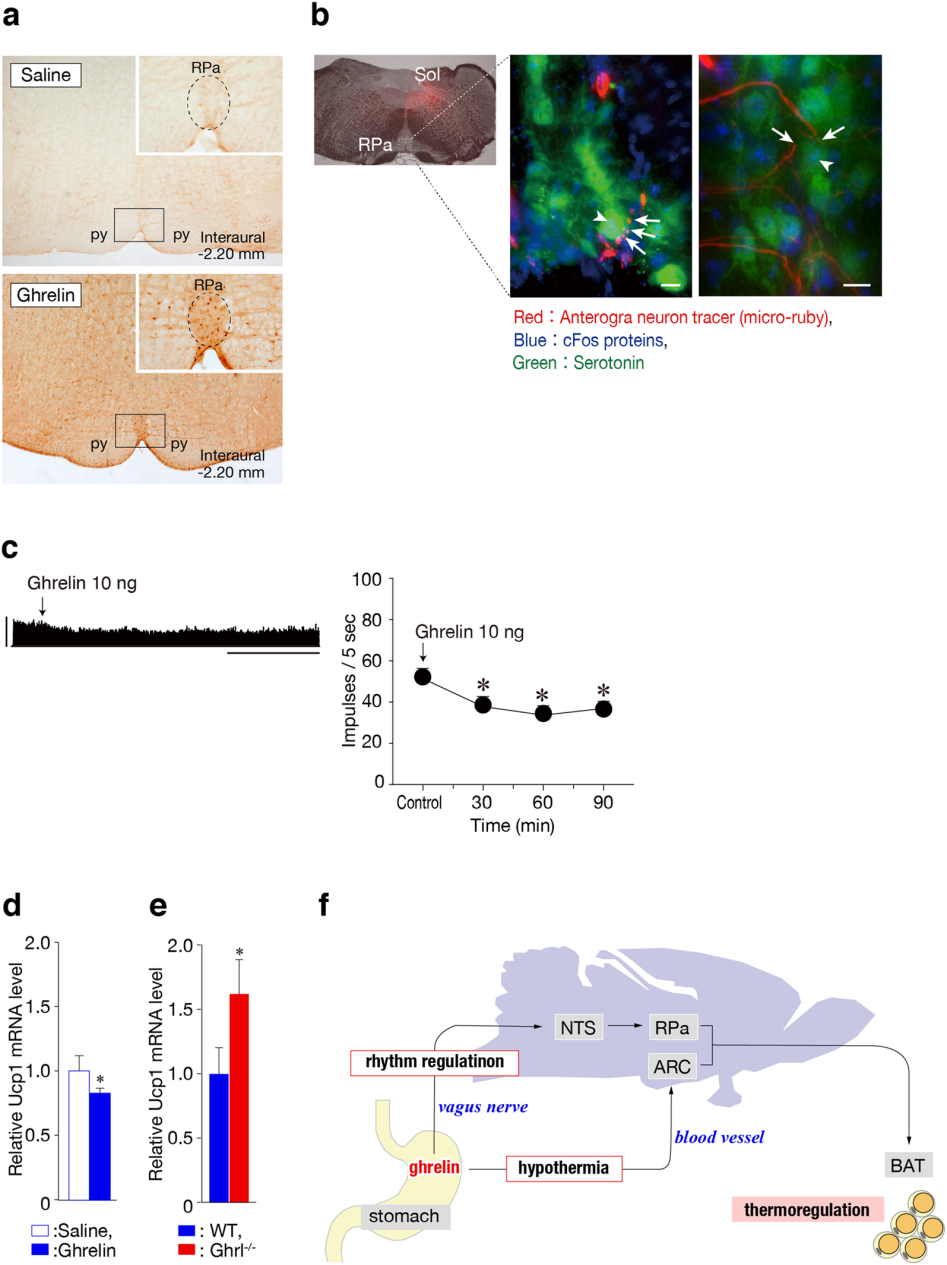
(**a**) c-Fos immunoreactivity in WT mice injected intraperitoneally with saline or ghrelin. The dotted ellipse indicates the rostral raphe pallidus nucleus (rRPa). Bars = 10 µm. (**b**) Neurons of the solitary nucleus projected onto ghrelin-activated neurons in rRPa. The nerve terminals of neurons from the solitary nucleus are indicated by arrows, and the nuclei of ghrelin-activated neurons to which these nerve terminals connect are indicated by arrowheads. The anterograde neuronal tracer Micro-ruby (red) was injected into the solitary nucleus. Serotonin neurons (green) identified immunohistochemically were used as markers for the rRPa. c-Fos immunoreactivity (blue) identified neurons activated after intraperitoneal injection with ghrelin. Bar = 10 μm. (**c**) Effects of ghrelin on BAT sympathetic nerve discharge. Intraperitoneal injection of ghrelin suppressed BAT sympathetic nerve activity. *P < 0.05 vs. value at 0 min. Data representing BAT sympathetic nerve discharge rates are shown in the upper figures. Vertical bar: 100 impulses / 5 s; horizontal bar: 30 min. (**d, e**) mRNA expression of the thermogenic-related gene *Ucp1* in BAT. The level of *Ucp1* mRNA was reduced 2 h after ghrelin administration to WT mice (d) and were more expressed in *Ghrl*^*−/−*^ mice than WT mice 36 h after fasting (**e**). *P < 0.05 (WT [n = 8] vs. *Ghrl*^*−/−*^ [n = 8] mice). (**f**) Schematic diagram of the effect of ghrelin on thermoregulation. This schematic diagram was created in Adobe Illustrator (ver. 25.0.1, https://www.adobe.com/jp/products/illustrator.html).

To identify the neural circuit responsible for ghrelin-mediated regulation of BT, we examined the distribution of c-Fos proteins, a marker of neuronal activity, throughout the brains of WT mice following intraperitoneal ghrelin injection. Although cFos expression was not induced by administration of saline in the rostral raphe pallidus nuclei of the medulla (rRPa), a thermoregulatory nucleus, cFos-positive cells were observed by administration of ghrelin (19.3 ± 2.2 cells). Therefore, it was found that peripherally administered ghrelin induces cFos expression of rRPa (Fig. 4a).

Since it has been reported that ghrelin secreted from the stomach transmits a signal to the solitary nucleus via the vagus nerve^8^, a reconfirmation experiment was conducted. When ghrelin was administered after excision of the vagal gastric branch of WT mice, almost no cFos-positive cells were observed in rRPa (0.3 ± 0.5 cells). Therefore, it was suggested that thermoregulation by ghrelin is also mediated by the vagal gastric branch. Then next, we investigated the neural projection from the solitary nucleus. As demonstrated by labeling with an anterograde neuron tracer, ghrelin-activated neurons in the rRPa received input from the solitary nucleus (Fig. 4b). This observation indicates that peripheral ghrelin transmits a neural signal via the vagus nerve to the solitary nucleus, and then to the medullary raphe regions.

In addition, electrophysiological studies revealed that intravenous administration of ghrelin significantly decreased sympathetic nerve activity in brown adipose tissue (BAT) (Fig. 4c). Because sympathetic nerve stimulation of BAT induces non-shivering heat production by increasing expression of *Ucp1* in BAT, we next measured *Ucp1* expression. The level of *Ucp1* mRNA in BAT decreased when ghrelin was administered, whereas *Ucp1* mRNA levels during fasting were higher in *Ghrl*^*−/−*^ mice than in WT mice (Fig. 4d, e). Next, we investigated the heat-producing ability of BAT in *Ghrl*^*−/−*^ mice. Intraperitoneal administration of the β3-adrenergic receptor agonist, CL316,243, increased BT in the same manner as in WT mice (Supplymentary Fig. 3a). In recent years, the regulation of mitochondrial fission associated with fasting has also been shown as a heat production mechanism different from UCP1. However, when observed 24 h after fasting, no fission occurred in the mitochondria of brown adipocytes of *Ghrl*^*−/−*^ mice (Supplymentary Fig. 3b). That is, the BAT of *Ghrl*^*−/−*^ mice itself has a normal ability of heat-production. Therefore, ghrelin suppresses non-shivering heat production by UCP1 by inhibiting BAT sympathetic nerve activity.

## Discussion

Mice can switch from a normal metabolic state into a hypothermic and inactive state when food availability is restricted. This phenomenon, which is called torpor, is thought to be analogous to hibernation. Torpor is an energy-conserving state characterized by suppressions of metabolic rate, heart rate, ventilation, and BT. In the torpor state, the metabolism shifts from carbohydrate oxidation to lipid metabolism.

In this study, we showed that *Ghrl*^*−/−*^ mice failed to decrease their BT and transit into torpor under low-energy states such as fasting and restricted feeding. A previous report showed that peripheral ghrelin deepens torpor bouts in NIH Swiss mice^9^. Therefore, ghrelin lowers BT and is required for the induction and maintenance of torpor in severe low-energy states.

However, Szentirmai et al*.* reported the opposite results, showing that *Ghrl*^*−/−*^ mice enter torpor, with BT dropping to near-ambient levels in response to fasting at 17 °C^10^. Moreover, they reported that when subjected to a more severe metabolic challenge, such as food deprivations in a cold environment, *Ghrl*^*−/−*^ animals entered precipitous hypothermia.

To resolve this discrepancy, we carried out a restricted feeding experiment using *Ghrl*^*−/−*^ mice. In the first half of the restricted feeding period, BT was higher in *Ghrl*^*−/−*^ mice than in WT mice. These results indicate that *Ghrl*^*−/−*^ mice consumed excess energy to maintain BT in the first half of the restricted feeding period using not only food energy but probably also energy stored in the body. However, in the second half of the restricted feeding period, when the food energy was restricted continuously, the BT of *Ghrl*^*−/−*^ mice dropped. These results indicate that *Ghrl*^*−/−*^ mice cannot maintain BT under continuous restricted feeding, even though the food-restricted condition used in our experiments did not induce torpor.

Uncontrolled BT in *Ghrl*^*−/−*^ mice was not due to a difference in energy intake; both WT and *Ghrl*^*−/−*^ mice consumed an equivalent amount of food under restricted feeding, as we demonstrated previously^11^. For animals to survive under restricted-feeding conditions, it is important that they maintain the balance between energy intake and energy loss. In other words, during restricted feeding, animals take in the food necessary to survive for a limited time, securing time for the exploratory behavior involved in seeking food, and then prevent waste of energy by decreasing BT during the remaining time. However, our results show that *Ghrl*^*−/−*^ mice could control feeding even in low-energy states, but had difficulty in regulating BT.

Previously, it was not clear whether ghrelin acts on the brain through the bloodstream or via the vagus nerve (16, 22, 23). Our findings clarify the role of ghrelin signal transduction in the BT regulation. Our conclusion is that ghrelin lowers BT mainly via the arcuate nucleus and stabilizes BT via the vagus nerve (Fig. 4f). Thus, ghrelin may cause different thermoregulatory responses through these two pathways.

In summary, our findings show that ghrelin plays an essential role in lowering BT and is required for the induction and maintenance of torpor during fasting. Under low-energy states, ghrelin may not only induce the sensation of hunger but also lower BT during the resting period, thus promoting adaptation to a low-energy state.

## Materials and methods

All the experiments were performed in accordance with ARRIVE guidelines and in accordance with relevant guidelines and regulations.

### Animals

Mice were generated by homologous recombination (Supplymentary Fig. 1). Briefly, the coding region of the *Ghrl* locus (from the ATG initiation codon to the termination codon) was precisely deleted from bacterial artificial chromosome (BAC)-based targeting vectors and replaced with a neomycin-selectable marker; the resultant construct was electroporated into embryonic stem (ES) cells. The presence of the construct in correctly targeted ES cells and derived heterozygous and homozygous mice was confirmed by PCR. After establishing germline transmission, mice were backcrossed against C57BL6/J mice to generate N6 breeding heterozygote pairs, which were used to generate homozygous null mice. Mice were housed under 12 h of light (7:00 a.m.–7:00 p.m.) per day at a constant temperature of 23 ± 1 °C. Animals were given ad libitum access to standard chow and water. All experiments with mice used male mice aged 10–12 weeks. The numbers used in each experiment are listed in Figure legends. All experimental procedures were reviewed and approved by the Ethical Committee for the Research of Life Science, Kurume University, and were in compliance with the Animal Research: Reporting in Vivo Experiments guidelines.

### Administration to mice

The following hormones and agonists were administered intraperitoneally in mice between 9:00 a.m. and 10:00 a.m. Rat ghrelin was kindly provided by Dr. Hiroshi Hosoda (National Cerebral and Cardiovascular Center). To investigate changes in BT after ghrelin administration, three doses of ghrelin were used: 1, 10, and 100 µg, in other ghrelin administration experiments, 1 µg of ghrelin was used. β3-adrenergic receptor agonist, CL316,243, was administered at 1 mg/kg to both WT mice and *Ghrl*^*−/−*^ mice.

### Implantation of mini-osmotic pumps

GHRP-6 (Sigma, St. Louis, MO, USA) was used as a ghrelin receptor agonist. To achieve continuous infusion of GHRP-6, we implanted subcutaneous Mini-osmotic Pumps (model 2002; Durect Co., Cupertino, CA, USA). The pumps delivered GHRP-6 at 42 ng/0.5 μl/h/g body weight for 14 days.

### Fasting experiment

WT and *Ghrl*^*−/−*^ mice were deprived of food for 48 h with free access to water, beginning at the end of the light phase (7:00 p.m.). Mice were given food again after 48 h.

### Restricted feeding experiment

Various behavioral and metabolic rhythms of mammals, including BT rhythms, are controlled in a 24-h cycle by an optical synchronous clock. However, when feeding in the light phase, it becomes possible to feed at a certain time of feeding, ignoring the optical synchronization clock. Ghrelin is a hormone that increases plasma concentration before feeding and starts feeding, and it is known that ghrelin concentration rises immediately before feeding when restricted feeding is performed. Therefore, in order to clarify the effect of ghrelin on the BT rhythm, it is necessary to perform it in the light phase. For this reason, Mice were restricted to a 4-h daily feeding period, beginning 3 h after lights-on (7:00 a.m.), for 11 consecutive days.

### Measurement of %rhythm

%rhythm was calculated based on the BT measurement data for 10 days. %rhythm is a chronobiological term for the coefficient of determination, i.e., the squared coefficient of correlation multiplied by 100 (%rhythm = r^2^ × 100); it represents the percentage of variation in the data that is explained by the fitted model.

### Implantation of the telemetric transmitter

Mice were anesthetized by intraperitoneal injection of ketamine and xylazine mixture (5 mg/kg; Dainippon Pharmaceutical, Osaka, Japan). A radiotelemetric device (TA10TE-F20; Data Sciences International, St Paul, MN, USA) was implanted to measure core BT. After the implant was placed within the abdominal cavity, the abdomen was closed. After implantation, mice were rapidly recovered with atipamezole. The diurnal rhythm of BT in mice was measured 3 weeks after the operation. During experimentation, a PhysiolTel-Receiver (model RPC-1; Data Sciences International) was placed under each animal’s cage to record core BT.

### Arcuate nucleus lesions

Using neonatal male mice (WT mice: n = 15, *ghrl*^*−/−*^ mice: n = 14), 10 μl of monosodium glutamate (MSG) (Sigma-Aldrich, St. Louis, MO, USA) was delivered at 4 mg/g on days 1, 3, 5, 7, and 9 after birth. Dead individuals (WT mice: 26.7%, *ghrl*^*−/−*^ mice: 28.6%) occurred in pups treated with MSG. At 4 weeks of age, the pups were weaned, bred and grouped by genotype. Since the body weight and food intake of WT and *ghrl*^*−/−*^ mice remained similar, it was considered that there was no difference in growth and feeding between the two groups by MSG treatment. Therefore, BT was measured using these individuals.

### Vagotomy

To transect the trunks of the subdiaphragmatic vagul nerves, the dorsal and ventral branches of the vagal nerves were dissected from the esophagus just the diaphragm after a left subcostal incision in the mouse. Each branch of the nerve was tied with surgical suture at two points and cauterized between the sutures.

### Measurement of hormones, blood glucose and free fatty acid concentration

*Blood glucose.* Blood glucose concentrations were measured using a self-administered blood measuring device (Glutestase R; Sanwa Chemical Research Institute Inc., Nagoya, Japan). *Free fatty acid.* Serum samples were subjected to Free Fatty Acid Assay Kit (Cell Biolabs, Inc. CA). *Ghrelin.* Plasma samples for quantification of ghrelin were prepared from whole blood mixed with EDTA-2Na (2 mg/ml) and aprotinin (500 kIU/ml). Immediately after plasma isolation, hydrogen chloride was added to samples at a final concentration of 0.1 N. Plasma samples were loaded onto Sep-Pak C18 cartridges (Waters, Milford, MA, USA), which were washed in 10% CH_3_CN/0.1% trifluoroacetic acid (TFA). Bound proteins were eluted in 60% CH_3_CN/0.1% TFA. The lyophilized eluate was subjected to ghrelin-specific ELISA (Active Ghrelin ELISA Kit and Desacyl Ghrelin ELISA Kit; Sceti, Tokyo, Japan). *Leptin and insulin.* Serum samples were subjected to Mouse/Rat leptin ELISA Kit (Morinaga Institute of Biological Science, Inc. Yokohama, Japan) for leptin and Ultrasensitive Mouse/Rat insulin ELISA Kit (Morinaga Institute of Biological Science, Inc. Yokohama, Japan) for insulin. All experiments proceeded according to the manufacturer's instructions. The genes and pathways whose expression fluctuations can be seen with this kit are as follows: Receptors: *Alpha Adrenergic Receptors* (Adra1a, Adra1b, Adra1d, Adra2a, Adra2b, Adra2c), *Hormone Receptors* (Adipor1, Adipor2, Insr, Lepr), *Nicotinic Receptors* (Chrna1, Chrnb1); Adenylate Kinases (Ak1, Ak2, Ak3); AKT & PI3 Kinase Signaling (Akt1, Akt2, Akt3, Pdpk1); Calcium / Calmodulin Signaling (Camkk1, Camkk2); AMP-Activated Protein Kinase: *AMP-Activated Protein Kinase Catalytic Subunits* (Prkaa1 (Ampk), Prkaa2), *AMP-Activated Protein Kinase Regulatory Subunits* (Prkab1, Prkab2, Prkag1, Prkag2, Prkag3); cAMP-Dependent Protein Kinases: *Protein Kinase A Catalytic Subunits* (Prkaca, Prkacb), *Protein Kinase A Regulatory Subunits* (Prkar1a, Prkar1b, Prkar2a, Prkar2b ); Protein Phosphatases: *Catalytic Protein Phosphatase Subunits* (Ppp2ca, Ppp2cb), *Non-Catalytic Protein Phosphatase Subunits* (Ppp2r1a, Ppp2r1b, Ppp2r2b, Ptpa); Autophagy (Atg13, Rb1cc1, Ulk1); Fatty Acid Metabolism (Acaca, Acacb, Cpt1a, Cpt1b, Cpt1c, Cpt2, Fasn, Gpam, Gpat2, Hmgcr, Lipe, Mlycd, Pnpla2); Glucose Metabolism (Gys1, Gys2, Pfkfb1 Pfkfb2, Pfkfb3, Pfkfb4, Slc2a4 (Glut4)); mTOR Signaling (Cab39, Mtor, Rptor, Stk11 (Lkb1), Strada, Stradb, Tsc1, Tsc2); Protein Synthesis (Eef2k, Eif4ebp1, Rps6kb1, Rps6kb2); Transcriptional Regulation (Crtc2, Cry1, Elavl1, Foxo3, Hnf4a, Ppargc1a (Pgc1alpha), Ppargc1b, Srebf1, Trp53 (p53)).

### Measurement of mouse circadian behavior

Twelve-week-old WT and *Ghrl*^*−/−*^ mice were individually housed in cages in the presence of an automated locomotor system (LOCOMO Sensor; Toyo Sangyou, Toyama, Japan). This system produces horizontal beams of infrared light (three beams in the x-dimension, two in the y-dimension) that span the width of the cage and encounter a detector on the opposite side of the cage. The number of times the mice crossed the beams was monitored by an on-line computer with a counter (CIF-32; MELQUEST, Toyama, Japan). During the LD cycle, the lights were on from 7:00 a.m. to 7:00 p.m. Period analysis of individual records was performed using the periodogram method (Muromachi, Tokyo, Japan).

### Real-time PCR of mRNA levels

cDNA was synthesized from total RNA (1 µg) extracted from mouse BAT using TRIzol Reagent (Invitrogen, Tokyo, Japan). Real-time PCR was performed on a PE Applied Biosystems PRISM 7000 Sequence Detection System (PE Applied Biosystems, Foster City, CA, USA). SYBR Green PCR Core Reagents (PE Applied Biosystems) were used for cDNA amplification. Each standard well contained pGEM-T Easy vector harboring the standard cDNA fragment. Relative mRNA levels were standardized against a housekeeping gene, mouse ribosomal protein S18 (*rps18*), to correct for any bias introduced during RNA isolation, degradation, or differential efficiency of reverse transcription. All primers for measurement of *Per2*, *Clock*, and *Ucp1* were purchased from Takara Bio (Kusatsu, Japan). AMPK signaling was measured using the PCR array method with RT^2^ Profiler PCR Array Mouse AMPK Signaling (Qiagen, Tokyo).

### Immunohistochemistry

Mice were anesthetized as described above and then perfused with 4% paraformaldehyde/PBS fixative. The brain was removed, then post-fixed with the fixative (4℃, overnight), and embedded in Tissue-Tek OCT-compound (Sakura Finetek, Tokyo, Japan). To investigate the effect of ghrelin on neural activity, we administered ghrelin (1 μg) to mice and perfused and perfusion fixation was performed 2 h later. Brain sections (40 µm) were stained with free-floating method using specific anti-c-Fos (Ab-5) (4–17) Rabbit pAb (1:20,000; Calbiochem: Sigma-Aldrich, Saint Louis, MO, USA). Single-labeling immunohistochemistry was performed according to the avidin biotinylated-HRP complex (ABC) method using an Elite ABC kit (Vector Laboratories, Burlingame, CA). Visualization of bound peroxidase was achieved by reaction for 5–10 min in a solution of 0.1 M Tris–HCl (TB; pH 7.4) containing 0.05% 3–3’-diaminobenzidine (DAB, Sigma-Aldrich, Oakville, ON, Canada). After a visualization reaction at the same time, it was confirmed that there was no staining on the control slides in which the primary antibody was omitted. To reveal the neuronal network involved in the ghrelin signaling cascade, we injected the anterograde neuronal tracer Micro-ruby (Invitrogen) into the solitary nucleus in WT mice. After 15 days, mice were perfused with 4% paraformaldehyde/PBS fixative and subjected to double-immunostaining. Brain sections (40 µm) were incubated with specific anti-serotonin (1:5000, rabbit polyclonal; Sigma-Aldrich, Saint Louis, MO, USA) as a marker for rRPa and anti–c-Fos monoclonal antibodies (8B5) (1:50; Assay Designs, Ann Arbor, MI, USA). The sections were observed by fluorescence microscopy (BZ-9000; Keyence, Osaka, Japan).

### Electrophysiology

BAT sympathetic nerve filaments isolated from adult male Wistar rats aged 10 weeks (CLEA Japan, Tokyo, Japan) were placed on a pair of silver wire electrodes to record afferent discharges. After ghrelin (10 ng) was administered intravenously, multiunit afferent nerve discharges were recorded and analyzed over a 90-min period.

### Transmission electron microscope

Both WT and *Ghrl*^*−/−*^ mice were divided into *ad lib* feeding and fasting 24 h groups, respectively, and each group was assigned 3 animals. Under deep anesthesia, the animals were perfused through the ascending aorta with physiological saline, followed by 2% glutaraldehyde (GA) in 0.1 M phosphate buffer (PB; pH 7.4). After perfusion fixation, brown adipose tissues were excised, cut into small pieces and further fixed in the same fixative for 24 h at 4℃. They were subsequently post-fixed with 1% osmium tetroxide (OsO4) in 0.1 M PB for 3 h at 4℃, dehydrated in a graded series of ethanol (70%, 80%, 90%, 95%, 100%, 30 min each), transferred to propylene oxide and embedded in epoxy resin by polymerizing for 48 h at 60℃. Ultrathin Sects. (80 nm thick) were cut with an ultramicrotome (EM UC7; Leica, Wetzlar, Germany) using a Diamond knife (Diatome, Biel, Switzerland) and mounted on grids. The sections were heavy metal staining with uranyl acetate and lead citrate and observed in a transmission electron microscope (H-7650, Hitachi, Tokyo, Japan) at accelerating voltage of 80 kV.

### Statistical analysis

Results are presented as means ± SEM for each group. Comparisons between groups were evaluated by ANOVA with a Bonferroni post-test. *P* values < 0.05 were considered statistically significant.

## Supplementary Information

Supplementary Information.

## References

[1] Kojima M (1999). Ghrelin is a growth-hormone-releasing acylated peptide from stomach. Nature.

[2] Ariyasu H (2010). A postweaning reduction in circulating ghrelin temporarily alters growth hormone (GH) responsiveness to GH-releasing hormone in male mice but does not affect somatic growth. Endocrinology.

[3] Asakawa A (2001). Ghrelin is an appetite-stimulatory signal from stomach with structural resemblance to motilin. Gastroenterology.

[4] Toshinai K (2001). Upregulation of ghrelin expression in the stomach upon fasting, insulin-induced hypoglycemia, and leptin administration. Biochem. Biophys. Res. Commun..

[5] Tschop M, Smiley DL, Heiman ML (2000). Ghrelin induces adiposity in rodents. Nature.

[6] Gavrilova O (1999). Torpor in mice is induced by both leptin-dependent and -independent mechanisms. Proc. Natl. Acad. Sci. USA..

[7] Shintani M (2001). Rapid publication ghrelin, an endogenous growth hormone secretagogue, is a novel orexigenic peptide that antagonizes leptin action through the activation of hypothalamic neuropeptide Y/Y1 receptor pathway. Diabetes.

[8] Date Y (2001). Ghrelin acts in the central nervous system to stimulate gastric acid secretion. Biochem. Biophys. Res. Commun..

[9] Gluck EF, Stephens N, Swoap SJ (2006). Peripheral ghrelin deepens torpor bouts in mice through the arcuate nucleus neuropeptide Y signaling pathway. Am. J. Physiol. Regul. Integr. Comp. Physiol..

[10] Szentirmai É, Kapás L, Sun Y, Smith RG, Krueger JM (2009). The preproghrelin gene is required for the normal integration of thermoregulation and sleep in mice. Proc. Natl. Acad. Sci. USA.

[11] Sato T (2008). Ghrelin deficiency does not influence feeding performance. Regul. Pept..

