# Supplementary material for: Thermoregulatory role of ghrelin in the induction of torpor under a restricted feeding condition
Source: Sci Rep. 2021 Sep 13;11:17954. doi: 10.1038/s41598-021-97440-y (PMC8438062; doi:10.1038/s41598-021-97440-y)
Supplement: Supplementary file 1 — Supplementary Information. [file 41598_2021_97440_MOESM1_ESM.pdf]

Title:

Thermoregulatory role of ghrelin in the induction of torpor under a restricted feeding condition

Authors:

Takahiro Sato[1], Kanae Oishi[1,4], Daisuke Koga[2], Takanori Ida[3], Yusuke Sakai[4], Kenji Kangawa[5] & Masayasu Kojima[1]

Affiliations:

[1] Molecular genetics, Institute of Life Science, Kurume University, 67 Asahi-machi, Kurume, Fukuoka 830-0011, Japan

[2] Department of Microscopic Anatomy and Cell Biology, Asahikawa Medical University, Asahikawa, Hokkaido 078-8510, Japan.

[3] Division for Searching and Identification of Bioactive Peptides, Department of Bioactive Peptides, Frontier Science Research Center, University of Miyazaki, Miyazaki 889-1692, Japan

[4] Institute of Animal Experimentation, Kurume University School of Medicine, 67 Asahi-machi, Kurume, Fukuoka 830-0011, Japan

[5] Department of Biochemistry, National Cerebral and Cardiovascular Center Research Institute, Suita 565-8565, Japan

Corresponding author:

Takahiro Sato, PhD & Masayasu Kojima, MD, PhD

Affiliation : Molecular genetics, Institute of Life Science, Kurume University

Address : 67 Asahi-machi, Kurume, Fukuoka 830-0011, Japan

TEL : +81(0)942-37-6313

FAX : +81(0)942-37-6319

E-mail : satou_takahiro@kurume-u.ac.jp, kojima_masayasu@kurume-u.ac.jp

Keywords:

ghrelin, torpor, thermoregulation

Supplementary Figure 1. Generation of *Ghrl*$^{-/-}$ mice and confirmation of genotype. (**a**) Schematic diagram of the murine wild-type (WT) ghrelin allele and targeting vector. Neo: neomycin-resistant gene. (**b**) Southern blot analysis of WT (+/+), heterozygous (+/-), and ghrelin-deficient (-/-) mice. (**c**) Plasma ghrelin levels in WT (n=8) and *Ghrl*$^{-/-}$ (n=8) mice, measured using active and des-acyl Ghrelin ELISA kits (SCETI, Tokyo, Japan). (**d**) Ghrelin mRNA expression in WT and *Ghrl*$^{-/-}$ tissues. GAPDH: glyceraldehyde-3-phosphate dehydrogenase. (**e**) Immunohistochemical analysis of ghrelin-positive cells in the stomach. Inset images show magnifications of the indicated rectangular regions. Bars = 200 μm. WT and *Ghrl*$^{-/-}$ mice.

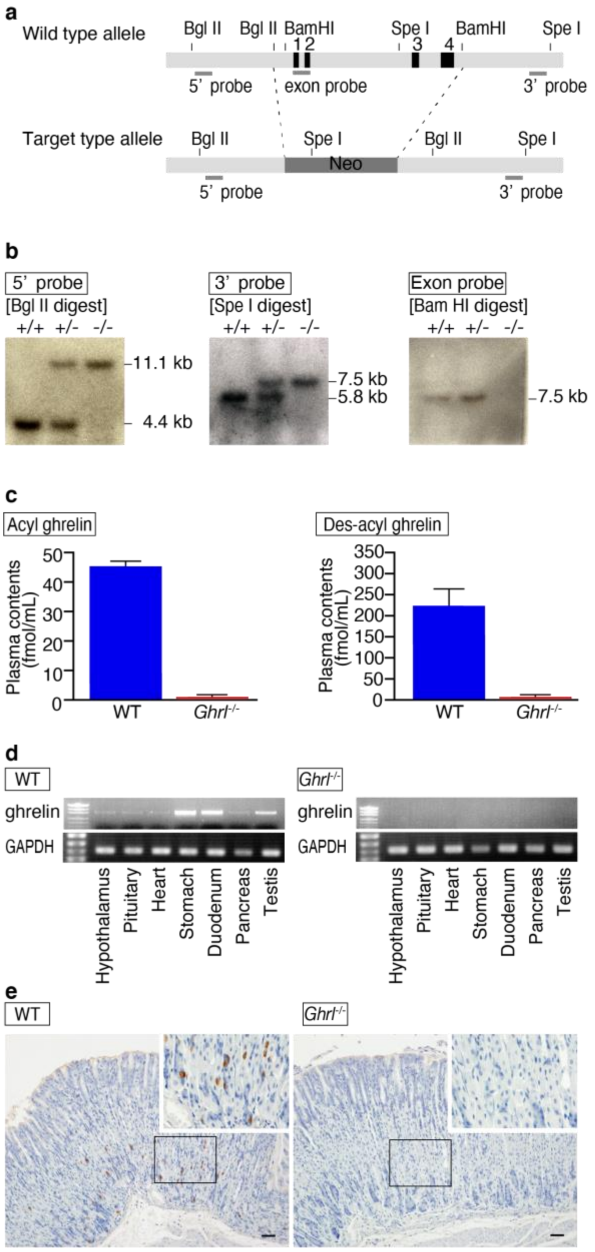

Supplementary Fig.1. Sato et al.

The following figure is a TIFF image of Supplementary Figure 1. (b).

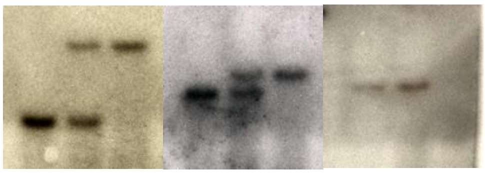

The following figure is a PSD image of Supplementary Figure 1. (d).

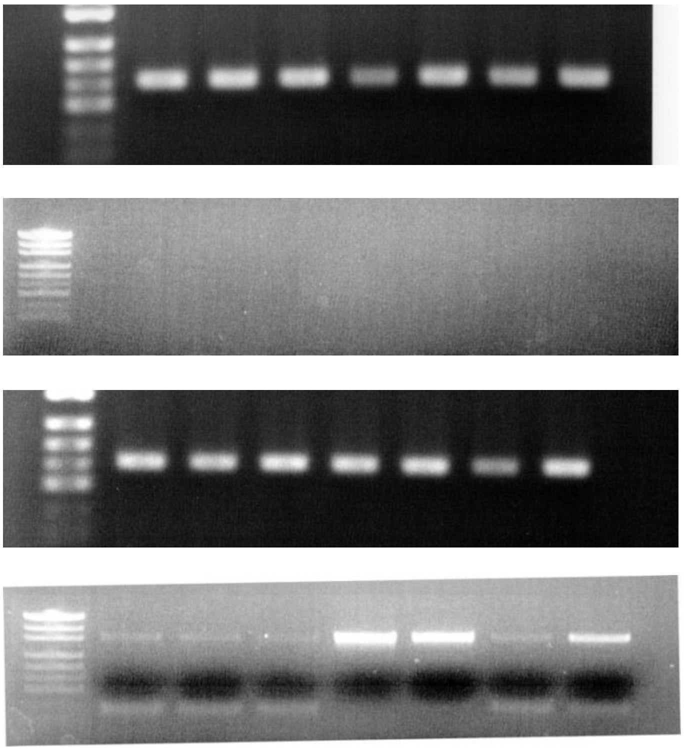

Supplementary Figure 2. Feeding regulator in *Ghrl*$^{-/-}$ mice. (**a, b**) Concentration of serum leptin (a) and insulin (b) with 48 h fasting. There was no difference in the content of any hormone between WT mice and *Ghrl*$^{-/-}$ mice. (**c, d**) Concentration of blood glucose (c) and of serum free fatty acid (FFA) (d) with 48 h fasting. There was no difference in the content of both parameters between WT mice and *Ghrl*$^{-/-}$ mice. (**e**) AMPK signaling pathway 24 hours after fasting analyzed by PCR array method in brain of WT mice and *Ghrl*$^{-/-}$ mice. The data are expressed as the ratio of GKO mice to WT mice for each gene expression level. No difference was found between WT mice and *Ghrl*$^{-/-}$ mice in all gene expression. All experiments were performed with WT [n=4] vs. *Ghrl*$^{-/-}$ [n=4] mice.

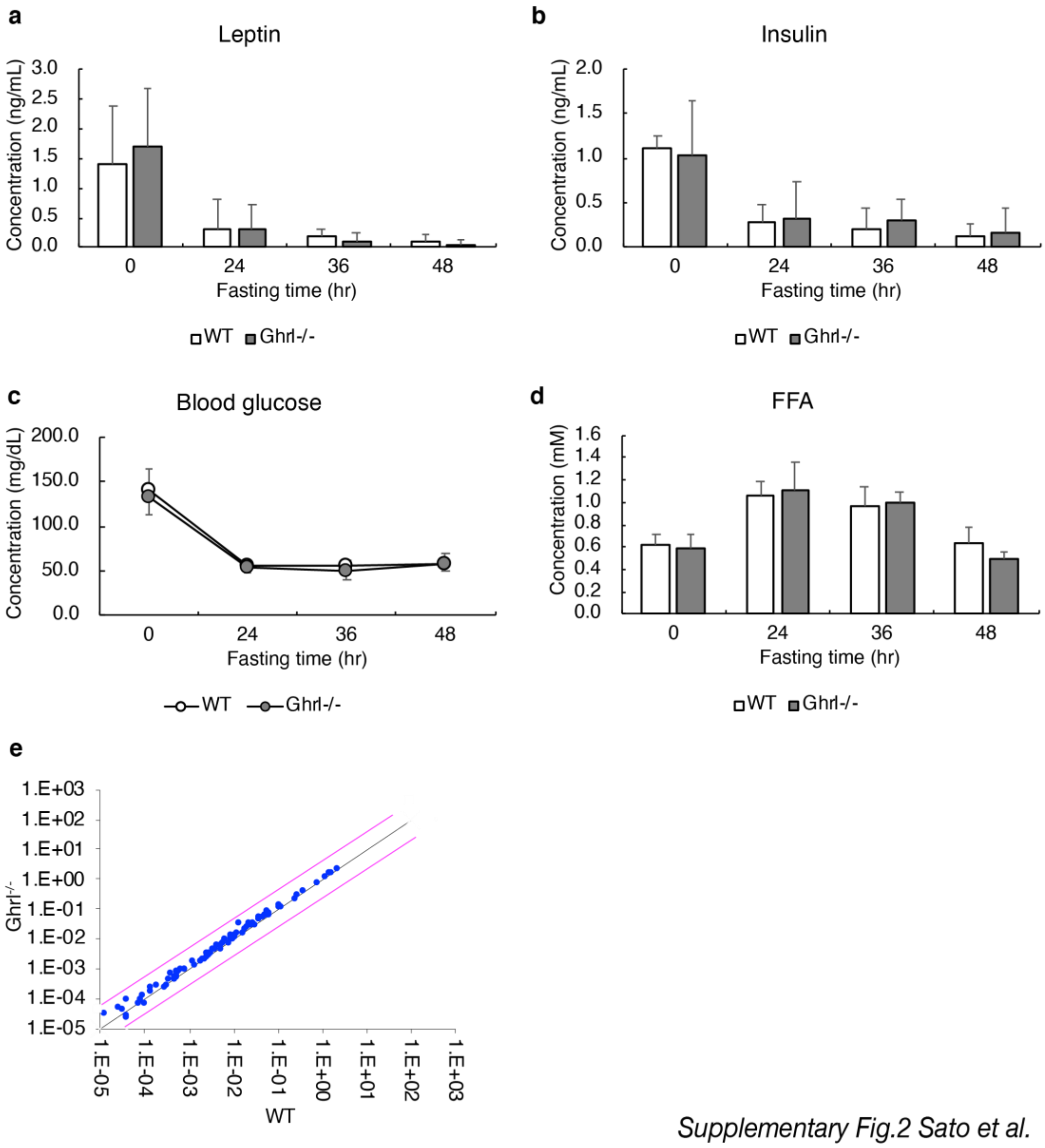

Supplementary Fig.2 Sato et al.

Supplementary Figure 3. Ability of heat production of BAT in *Ghrl*$^{-/-}$ mice. (**a**) Changes in BT of *Ghrl*$^{-/-}$ mice after β3-adrenergic receptor agonist, CL316,243, administration. WT mice [n=3] vs. *Ghrl*$^{-/-}$ mice [n=4]. (**b**) Electron microscopy of mitochondria of brown adipocytes during 24 h fasting of WT mice and *Ghrl*$^{-/-}$ mice. FD: fat depot, M: mitochondrion, N: nuclear. Bar = 1 μm.

a

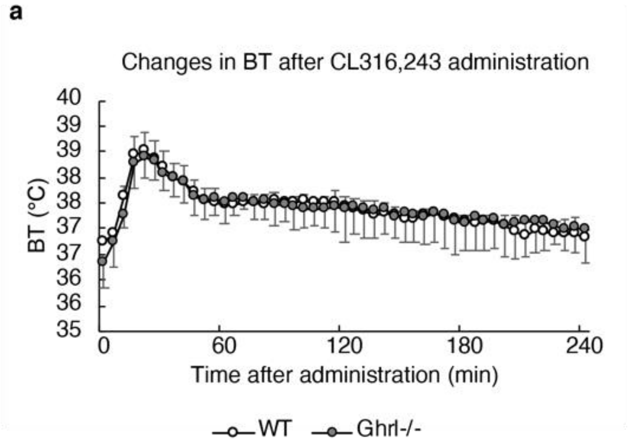

b

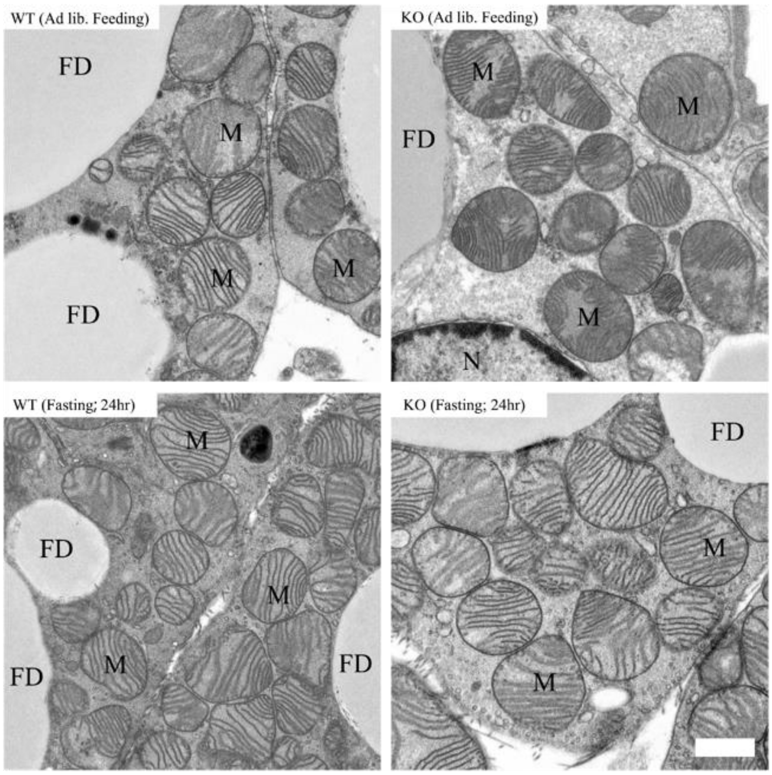

Supplementary Fig.3 Sato et al.
